# Mid-Infrared Spectroscopy as a Valuable Tool to Tackle Food Analysis: A Literature Review on Coffee, Dairies, Honey, Olive Oil and Wine

**DOI:** 10.3390/foods10020477

**Published:** 2021-02-22

**Authors:** Eduarda Mendes, Noélia Duarte

**Affiliations:** Research Institute for Medicines (iMED.Ulisboa), Faculty of Pharmacy, Universidade de Lisboa, Av. Prof. Gama Pinto, 1649-003 Lisbon, Portugal; ermendes@ff.ulisboa.pt

**Keywords:** mid-infrared spectroscopy (MIR), FTIR, ATR, food adulteration, food authenticity, chemometrics

## Abstract

Nowadays, food adulteration and authentication are topics of utmost importance for consumers, food producers, business operators and regulatory agencies. Therefore, there is an increasing search for rapid, robust and accurate analytical techniques to determine the authenticity and to detect adulteration and misrepresentation. Mid-infrared spectroscopy (MIR), often associated with chemometric techniques, offers a fast and accurate method to detect and predict food adulteration based on the fingerprint characteristics of the food matrix. In the first part of this review the basic concepts of infrared spectroscopy, sampling techniques, as well as an overview of chemometric tools are summarized. In the second part, recent applications of MIR spectroscopy to the analysis of foods such as coffee, dairy products, honey, olive oil and wine are discussed, covering a timespan from 2010 to mid-2020. The literature gathered in this article clearly reveals that the MIR spectroscopy associated with attenuated total reflection acquisition mode and different chemometric tools have been broadly applied to address quality, authenticity and adulteration issues. This technique has the advantages of being simple, fast and easy to use, non-destructive, environmentally friendly and, in the future, it can be applied in routine analyses and official food control.

## 1. Introduction

Food fraud and determination of food authenticity have been subjects of utmost importance for food industry, causing major concerns among food manufacturers, regulatory agencies and consumers, particularly after the melamine scandal in China in 2007 and the horse meat scandal in Europe in 2013 [1]. One of the most common type of food fraud is the intentional and economically motivated adulteration of foods, which can include the replacement of a high-value nutrient with a low-value one, the dilution of a high value liquid ingredient with a cheaper liquid, the addition of foreign materials in order to enhance food quality or organoleptic attributes, or hiding the low quality of food ingredients or products [1]. The adulteration of food products not only compromises its authenticity and reduces product quality, but also may seriously jeopardize consumer’s health. Although all foods can be susceptible of potential adulteration, there are certain products that are considered more prone to fraudulent acts, particularly if they are produced and supplied in complex market chains, if they are considered as luxurious food commodities, or on the other hand, if they have low margins of profit. These products include spices, honey, olive oil, wine, milk and dairy products, coffee and tea, among others [2,3].

Over the years, several analytical methods have been employed to detect food adulteration and determine its authenticity. These techniques include target analyses that identify specific compounds and assess if they are within the limit stated on the label or established by law, and non-target analyses that afford a fingerprint of the whole sample, which can then be used to evaluate its authenticity or detect a possible adulteration [1,4,5]. The most commonly used techniques are physicochemical analytical methodologies, gas and liquid chromatography frequently hyphenated with mass spectrometry, immunochemical and DNA based techniques, isotope ratio, elemental analysis and spectroscopic techniques that include UV-Visible, infrared, Raman and nuclear magnetic spectroscopy (NMR) [4,5]. All of these techniques have their own pros and cons. For example, classical physicochemical methods are time consuming, some of them involve many analytical steps and require large volumes of organic solvents that generate toxic wastes. Although very selective and specific, the modern chromatographic methodologies, NMR and mass spectrometry require the acquisition and maintenance of very expensive instrumentation and highly qualified laboratory technicians, and frequently also involve the need of very complex sample pre-treatment [5].

Spectroscopic techniques, in particular near-infrared (NIR) and mid-infrared (MIR) spectroscopy, have been increasingly and broadly used in food analysis [1]. Moreover, in the last two decades, hyperspectral imaging has also emerge as a promising technology [6,7]. Some advantages of these non-destructive techniques include the high speed of analysis, minimal or no sample preparation and no waste generation. Hyperspectral imaging has also the advantage of simultaneously integrating both spectral and imaging data to obtain chemical, spatial and multi-constituent information about a sample. With this technique, single or multiple images can be obtain at selected wavelengths in the UV-VIS, NIR or MIR range, giving the ability to reveal particular features directly correlated with quality [8]. However, both spectroscopic and imaging techniques produce a large amount of spectral data that always implies the association of chemometric tools to extract the most interesting information [2,9].

This review aims at giving an overview of the recent applications of MIR spectroscopy for the determination of adulteration and authenticity of five foodstuffs that are considered more prone to fraudulent acts, namely, coffee, dairy products, honey, olive oil and wine. A brief introduction to the basic concepts of IR spectroscopy, IR sampling techniques and chemometric tools is provided. Finally, several approaches concerning the future implementation of MIR spectroscopy in food industry is also discussed.

The literature search on planned topics was carried out from February to March 2020 using the Web of Science, ScienceDirect and PubMed databases. The suitable combination of keywords and truncation were selected and adapted for each database. Only reviews and research articles published in English in a ten-year timespan were considered. Some official websites were also accessed. All references were managed using the Mendeley desktop software and duplicates were excluded. The authors individually screened the articles for their relevance on the studied topics.

## 2. Basic Concepts of Infrared Spectroscopy

**Figure 1:**
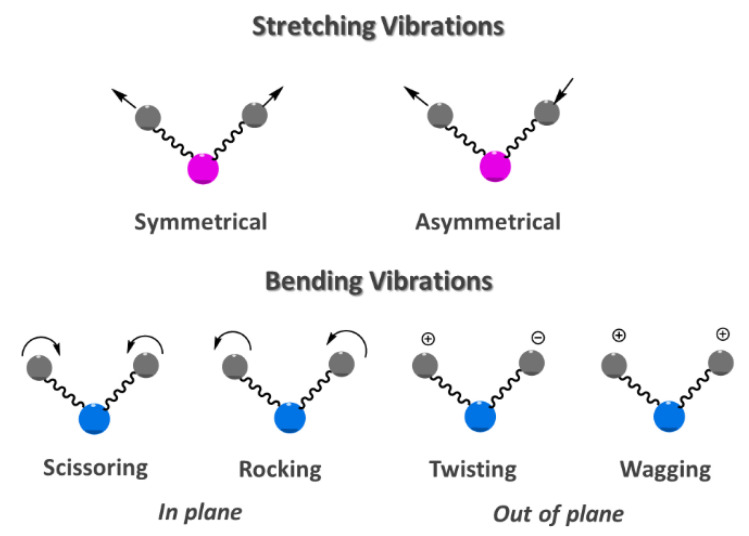
Major stretching and bending vibrational modes.

Infrared (IR) spectroscopy is based on the absorption or reflection of the electromagnetic radiation that ranges on wavelengths (λ) between 0.78 and 1000 µm. Typical units of frequency used in IR spectra are wavenumbers (cm^−1^) that are related with wavelength (cm) in a reciprocal way. The infrared region of the electromagnetic spectrum is generally split in three smaller zones, namely, near-IR (12800–4000 cm^−1^; 0.78–2.5 µm), mid-IR (4000–200 cm^−1^; 2.5–50 µm) and far-IR (200–10 cm^−1^; 50–1000 µm) [10]. In a polyatomic molecule, the absorption of mid IR radiation causes two fundamental vibrational modes, the stretching and bending vibrations (Figure 1) [11].

MIR spectra present several absorbance bands due to fundamental transitions. Spectra can be analyzed looking at two major regions, the functional group (4000–1300 cm^−1^) and the fingerprint (1300–600 cm^−1^) regions, therefore providing a molecular fingerprint. The functional group region can be further subdivided in specific zones representative of functional groups and includes the X–H stretching (4000–2500 cm^−1^; X=C, N, O or S), the triple-bond (2700–1850 cm^−1^), and the double-bond (2000–1500 cm^−1^; C=C, C=N, C=O) stretching regions [12]. The fingerprint region is characterized by a complex group of bending vibrations and shows many bands, frequently overlapped, that are specific to the molecular structure of the sample [11]. In spite of its complexity, the fingerprint region has been used for detection of adulteration and authentication of various food products [9].

The absorptions observed in the NIR region arise from overtones or combination of the fundamental bands in the MIR region, particularly those resulting from the molecular vibrations of hydrogen bonds such as –C–H, –S–H, –N–H, and –O–H functional groups. These bands are less intense, and frequently overlapped resulting in poorly resolved and very complex spectra [13,14]. A major disadvantage of NIR spectroscopy has been the difficulty of extracting the information, and the interpretation of the large amounts of spectral data. These limitations have been overcome by the increasing development of chemometric tools and nowadays, NIR spectroscopy is considered as an important analytical technique in food, phytoanalysis, biomedical applications and polymers studies, among others [9,14].

The original MIR spectrophotometers were dispersive equipment that used diffraction gratings as monochromators. These instruments were replaced by Fourier Transform infrared (FTIR) spectrophotometers that use the Michelson-type interferometer instead of the diffraction grating, coupled to microprocessors that applies the FT mathematical operation for obtaining the spectrum. When comparing with the old dispersive spectrophotometers, FTIR has several advantages including better sensitivity, resolution and speed of analyses [9].

**Figure 2:**
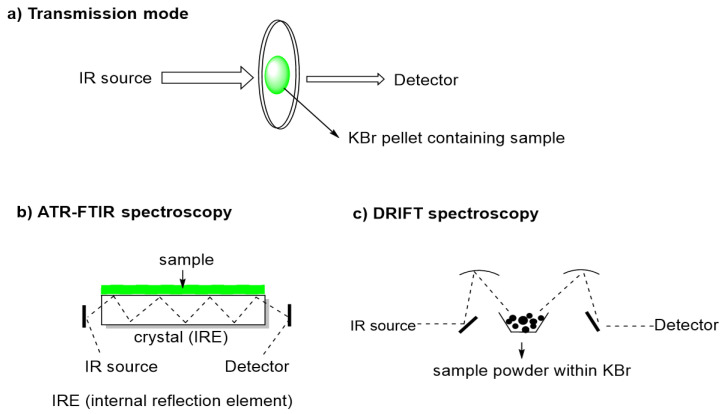
Sample presentation techniques for vibrational spectroscopy.

FT-MIR and sampling instrumentation have been evolving over the years. There are three commonly used sample presentation techniques for vibrational spectroscopy: transmission, attenuated total reflection (ATR), and diffuse reflectance (DRIFT) (Figure 2) [15,16]. Transmission spectroscopy is the oldest and most basic technique and, in the case of liquid samples, these are placed in fixed-length transmission cells. For solids, Nujol Mull (Nujol—a liquid paraffin oil) or alkali halide (KBr) pellets, were the standard options [2].

Reflection techniques are based on the reflection of the IR beam after contacting with the sample and receive distinct designation depending on reflection process such as attenuated total reflectance (ATR) also called multiple internal reflectance (MIR) and diffuse reflectance by infrared Fourier transform spectroscopy (DRIFT). These reflectance techniques operate with different optical properties and, as advantage, they do not require the traditional sample preparation used for transmission FTIR spectroscopy. J. Fahrenfort was the first to demonstrate the usefulness of the ATR method [17]. In this reflection technique an IR beam is directed onto an optically dense crystal (ZeSe, ZnS, Ge or diamond) with a high refractive index at a certain angle [2]. The internal reflectance creates an evanescent wave that extends beyond the surface of the crystal into the sample held in contact with the crystal. In regions of the IR spectrum where the sample absorbs energy, the evanescent wave will be attenuated and the detector records the attenuated IR beam as an interferogram signal, which can then be used to generate an IR spectrum [18]. The use of total reflectance attenuated by multi-reflectance, in which the beam is reflected internally many times, was developed by Harrick, who design ATR cells for commercial use [2,17]. The main advantage of ATR sampling comes from the very thin sampling path length and depth of penetration of the IR beam into the sample, in general of 0.1–5 micrometers, which makes sampling in aqueous solutions possible and more importantly, practically requires no sample preparation [17,19]. ATR is today the most widely used FTIR sampling tool, namely in food analysis because it allows fast and simple sampling.

Diffuse Reflectance Infrared Fourier Transform spectroscopy (DRIFT) can provide a convenient means of examining finely powdered or highly scattering solid samples but it is most commonly used for near infrared (NIR) spectroscopy applications [20]. It is used for measurement of fine particles and powders, as well as rough surfaces and requires simpler sample preparation compared with transmission FTIR. In DRIFT spectroscopy, the IR beam penetrates the analytical sample to a certain depth, and is then re-emitted from the sample and focused by a mirror onto the detector. The resulting DRIFT spectrum is similar to that obtained by transmission FTIR technique, although the former is more dependent on physical characteristics of samples like absorptivity and reflectance. The DRIFT preparation technique still requires samples to be mixed with KBr, but avoids the need for pelleting with a hydraulic press. This technique can result in increased resolution of the spectra and reduced interference from water bands compared to transmission techniques. The DRIFT applications in food analysis are limited, but can be found in some reports on quality of coffee [15,21,22,23], of rice [24], and more recently, sugars in mangoes [16].

## 3. Chemometric Tools Used in Data Analysis

Food is a very complex matrix that contains a high number of components giving rise to a multitude of spectral information and large data sets. Consequently, fast statistic and mathematical analyses are needed to fully understand all the complexity of data, as well as the usefulness and the rigorous control of the integrity of the information obtained [25].

The classic univariate statistic methods, such as analysis of variance (ANOVA), are focused on the reductionist approach (e.g., one variable at times), and food compounds or properties are analyzed independently of the entire food matrix. Conversely, modern chemometric methods are multivariate analysis approaches that allow the treatment of multidimensional and complex data sets, sometimes with only subtle differences among all the data sets, revealing properties that are important through their various interferences and interactions in the whole food matrix [25,26]. Multivariate methods can be applied either to qualitative and quantitative analysis, letting relevant information to be extracted from complex data, allowing the creation of empirical models that could be used to perform exploratory studies and describing important characteristic of samples or predictive analyses on new samples [27].

Chemometric tools can be divided in two types of pattern recognition methods: unsupervised and supervised [26]. In the unsupervised methods, the classification of the dataset is made regarding the differences and similarities among the samples, providing information about the relationship between samples, between variables and between samples and variables, without using any previous information [28]. The most common tools for unsupervised pattern recognition are Cluster Analysis (CA), Hierarchical Cluster Analysis (HCA) and Principal Component Analysis (PCA). These tools reveal clusters without taking in consideration the number and specific type of classes. The results are usually presented in the form of a graphical representation showing the grouping of samples (clusters). PCA is a commonly used tool that could reduce a complex spectra data set to a small number of no correlated variables, while retaining trends and patterns. For each variable a score for each sample is calculated. Graphical display of these scores reveals patterns, gathering similar samples close to each other [19,29]. Moreover, it is also possible to disclose the existence of potential outliers, samples that are apart from the main group and should be rejected [28]. The supervised models, also known as classification models, identify an unknown pattern as a member of a predefined class. These models are intended to find mathematical patterns that are able to recognize samples as members of specific and already known classes, assigning samples to them. New unknown samples could be individually assigned into these known classes depending on the experimental results [26,27]. Examples of these methods include, Linear Discriminant Analysis (LDA), Fisher Discriminant Analysis (FDA), Quadratic Discriminant Analysis (QDA), Regularized Discriminant Analysis (RDA), k-Nearest Neighbor (KNN), Support Vector Machine (SVM) and Partial Least Squares Discriminant Analysis (PLS-DA). Several prognostic multivariate models have also been used, such as, Partial Least Squares Regression (PLSR) and Artificial Neural Networks (ANN) [26]. Data fusion (DF) is a strategy that is being increasingly developed. It consists of combining and analyzing complex data obtained from several instrumental methods, in order to synergistically gather complementary information and more reliable results. Initially, data fusion comprised the use of two complementary analytical methods, such as MIR and UV-Visible spectroscopy, or the application of gas or liquid sensor devices with NIR or MIR spectroscopy. More recently, the combination of data from three, four and even five techniques has been reported [28,30]. There are three levels of data fusion: low-, mid- and high-level data fusion [28,31]. In the low-level approach, raw data from two or more techniques are simply concatenated into a matrix, with or without the pre-processing of data. The matrix is then used to assess a single model that gives the final classification [30]. In mid-level data fusion some relevant raw variables are firstly selected from each data source, and fused thereafter. The resulting cluster is then used for multivariate classification and regression. In the high-level data fusion, regression or classification models are previously calculated for each data set and the obtaining results are then fused [28,30]. Ultimately, DF allows the complementary information to be evaluated, strengthening the conclusions obtained or hidden information to be unveil [30]. An exhaustive description of chemometric tools is beyond the scope of this review and the interested reader should consult other publications [26,28,29,32,33].

## 4. Application of FT-MIR to Food Analysis

### 4.1. Coffee

Coffee is a commodity of major economic importance, being the second most consumed and one of the most appreciated beverages in the world. Although more than eighty *Coffea* species are known, only two, *Coffea arabica* and *Coffea canephora* (var. Robusta) have economic importance [34]. Accounting for the livelihood of around 125 million people around the world, it is currently produced in about 80 countries of four continents, mainly in equatorial areas of Southeast Asia, Africa, India, and America [32]. According to International Coffee Organization, the leading coffee-producing countries are Brazil, Vietnam, Colombia, Indonesia, Ethiopia, India, Uganda and Mexico [32]. Brazil is the largest coffee producer in the world accounting for 70% of total Arabica variety. Vietnam has been recognized as the world’s largest exporter of Robusta coffee (40% of the total production) followed by Indonesia [33,35].

Arabica and Robusta varieties have different features including their geographical distribution, physical aspects, chemical composition and post-harvest processing, which will influence the quality and organoleptic characteristics of coffee beverage. *Coffea arabica* plants grow well at higher elevations and mild temperatures than *Coffea robusta* and accounts for approximately 70% of the global coffee production [36].

Green coffee beans are mainly composed of carbohydrates (59–61%), lipids (11–17%), proteins (10–16%), phenolic compounds (6–10%), minerals (4%), fatty acids (2%), and the alkaloids caffeine (1–2%) and trigonelline (1%). During the roasting process, the change of lipids, minerals, fatty acids, caffeine and trigonelline is small, while carbohydrates (38–42%), proteins (8–14%) and phenols (3–4%) are reduced [37]. Moreover, polymeric compounds called melanoidins and more than 900 volatile compounds are also formed during the roasting process, being responsible for the typical flavor and aroma of the beverage. Arabica coffee has superior organoleptic characteristics and quality and therefore, it is more expensive. On the other hand, Robusta coffee has a more wooden and earthy flavor, sometimes with strong and pronounced bitterness [38]. This variety contains more soluble solids and higher amounts chlorogenic acids, trigonelline and caffeine [39]. Commercial coffee is made from Arabica or Robusta varieties or their blends in different percentages.

The quality of coffee beans depends on good agronomic practices and post-harvest operations, such as fruit selection, processing, drying and storage. Additionally considered of utmost importance is the roasting process because the high temperatures promote physical and chemical changes that have major effects in color, taste and aroma. The most frequently accepted quality factors are bean size, moisture content, number of defects, aspect and chemical composition of green and roasted beans, the roasting process and cup preparation [38,40].

Due to high market value and commercial importance, the adulteration of coffee is very common, particularly for ground coffee. It can include the substitution of *C. arabica* more expensive beans by *C. canephora* or defective beans, as well as the dilution with several cheaper products such as coffee husks, spent coffee grounds, barley, wheat, corn, chicory and cocoa or soya beans [41,42,43]. Increasingly important for the global coffee trade market is the geographical origin and geographical indication, as consumers usually search for coffee of high quality from beans of known origins. Moreover, there are significant differences in coffee price depending on country and region of origin [44]. The authenticity of coffee regarding its country origin is rather important for producers, traders and consumers, and a valid certificate of origin, under the International Coffee Agreement 2007, shall cover every export of coffee by an exporting country [45,46]. This certification protects producers against counterfeiting and increases the strength of production chains and the economic development through international market competitiveness.

Currently, there is no validated method to establish the geographical origin or the presence of adulterants in coffee samples. Several analytical techniques have been attempted, such as chromatographic methods (GC-MS, HPLC-MS or HPLC-DAD) or spectroscopic techniques (NIR, Raman, MIR or NMR), most of them coupled to chemometric methods [41,42]. Application of MIR-FT spectroscopy to the analysis of coffee is presented in Table 1.

FTIR spectroscopy associated with radial-basis function network (RBF), an artificial neural network (ANN) that is widely used for pattern classification, was successfully employed by Link et al. for the geographic and genotypic classification of Arabica coffees [47]. This chemometric procedure was able to classify the samples, both geographically (100% correct classification) and genotypically (94.44%), exhibiting a superior performance when compared with other methods such as Soft Independent Modeling of Class Analogies (SIMCA) that have also been applied to the same test set [48].

A study by Obeidat et al. [46] discriminated among samples of green coffee beans of five different origins (Brazil, Colombia, Ethiopia, Kenya, and Yemen) using FTIR spectra, without any preliminary treatment of the samples. FTIR spectra were collected in the 600–4000 cm^–1^ range and two regions, 1775–1500 cm^–1^ and 3030–2750 cm^–1^ were found to be important for analysis. However, the visual analysis of IR spectra is rather difficult since all of them are very similar. Therefore, PCA was used to successfully categorize each coffee type, concluding that bands at 2850, 2920, and 1745 cm^–1^ are the most important in distinguishing the origin of the coffee samples.

Medina et al. [49] compared the ability of ^1^H-NMR, ATR-MIR, and NIR spectroscopic techniques to discriminate *C. arabica* from *C. robusta* as well as Colombian coffees from other samples collected on fourteen neighbor and worldwide countries. PCA was used to test data quality and identify possible outliers, whereas PLS methods were used as classifiers. ^1^H-NMR and ATR-MIR showed similar ability to distinguish Colombian coffee samples, but weak results were obtained for NIR. This assumption is not in accordance with the study reported by Bona et al. [50], which also assessed the potential of MIR and NIR for geographical classification of twenty Arabica coffee genotypes. Using Support Vector Machines chemometric algorithm, NIR showed a superior performance when compared to MIR, achieving a sensitivity and specificity of 100%. It was proposed that the worst results obtained with MIR may be due to the use of KBr pellets instead of ATR sampling procedure, which is generally reported to have better outcomes. Nevertheless, it can be concluded that IR spectroscopy could be effective for geographic authentication of coffee.

The presence of defective coffee beans (sour, black and immature beans) decreases the quality of roasted and ground coffee, and ultimately also decreases the quality of the final beverage, as they are associated with sour, oniony and astringent tastes. The main method to separate defective from non-defective beans is by electronic color sorting or by sieving, which precedes the roasting process. Craig et al. evaluated the potential of FTIR in association with multivariate statistical analysis (PCA, LDA) to distinguish defective and non-defective green Arabica coffee beans [22,51]. The sample lots were divided in black, sour (light and dark colored), immature and non-defective beans. Three FTIR sampling techniques were compared, including the traditional transmittance method (KBr discs) and reflectance methods (DRIFT and ATR). All the transmittance and reflectance spectra were qualitatively similar, although some absorbance intensity differences could be observed in the 1800–800 cm^−1^ range. PCA analysis of the KBr transmission spectra was able to define three sample sets, non-defective, immature and black/dark sour. Using DRIFT, it was possible to successfully recognize four major clusters: non-defective, immature/light sour, dark sour, and black. It was also possible to establish the spectral ranges that most contribute to the sample clustering, including the bands at 2980–2850 cm^−1^ and 1560–800 cm^−1^ (immature/light sour samples), 1700–1570 cm^−1^ (non-defective beans), 3100–3000 cm^−1^ and 1980–1760 cm^−1^ (dark sour beans), and 2000–1985 cm^−1^ (black beans). Contrary to transmission and reflectance techniques, ATR only provided the separation of samples in defective and non-defective coffees not allowing the grouping of defective beans according to the type of defect [22,51]. The same research group also compared the performance of DRIFT, ATR and NIR to discriminate between defective and non-defective roasted Arabica coffee beans [52,53]. Samples were divided in five lots, non-defective, immature, black, sour (light and dark colored), and roasted at 220, 235 and 250 °C. PCA, LDA and a statistical approach named Elastic Net were used as chemometric methods. Principal Components Analysis of DRIFT spectra grouped the samples into four clusters: non-defective, black, dark sour and light sour, with immature beans scattered among the sour samples. On the other hand, LDA classification models based on absorbance bands at eight wavenumbers (2924, 2852, 1743, 1541, 1377, 1076, 910 and 816 cm^−1^) allowed, besides the above-mentioned groups, the differentiation of the immature beans. Therefore, a clear separation between defective and non-defective beans was achieved [52]. The classification models based on elastic net statistic model exhibited high percentage of correct coffee classification (100% for calibration and above 94% for validation) [53].

Brondi et al. [58] studied the adulterations of roasted and ground *Coffea arabica* L. by corn using two methodologies, ATR-FTIR and differential scanning calorimetry, associated to PCA in order to establish the clusters, and PLS approach aiming at quantify the level of adulteration [58]. Green coffee beans were roasted at three different temperatures from light to dark roasts, grounded and mixed with ground roast corn in a range of 0.5 to 40% (m/m). Although there were no differences between the spectra of three levels of roasting, significant differences were found between the spectra of roasted coffee and corn. PCA approach allowed the recognition of pure and adulterated samples even in concentrations lower than 1%, although it was not able to distinguish the different roasting levels. PLS models exhibited a good correlation between the values of estimated and reference concentrations with root mean square error of cross-validation of 2.1%. Differential scanning calorimetry analysis presented similar results when compared to FTIR, but it has been mostly used to qualitative analysis. However, authors considered that the two techniques could be used as complements for adulteration detection and quantification purposes [58].

A method to quantify Robusta coffee in Arabica coffee blends using ATR-FTIR and a PLS model was developed by Correia et al. [59]. To build this model, the most important wavelengths bands were those around 2900 cm^−1^, 1750 cm^−1^ and above 1000 cm^−1^. A good correlation was found between the predicted and the reference values with coefficients of determination for cross-validation and prediction of 0.9635. In addition, ESI-FT-ICR mass spectrometry was also used to investigate the chemical profiles of Robusta and Arabica coffees [59].

Reis et al. reported a series of studies to assess the potential of DRIFT for analysis of the adulteration of pure roast Arabica coffee with coffee husks, spent ground coffee, corn and barley, despite the roasting conditions [16,22,59]. PCA and LDA were applied to find out if each one of the studied adulterants, as well as adulterated coffee samples (at adulteration levels ranging from 1 to 66 g/100 g) could be discriminated from pure roasted coffee [15]. A comparative evaluation of all spectra allowed the identification of absorption bands in two major wavelength ranges, 3000–2800 cm^−1^ and 1800–700 cm^−1^, and their correlation with the presence of some important constituents in the different matrices that consequently, contributed most to the discrimination of samples. Sharp bands at 2925 and 2852 cm^−1^ were partly assigned to unsaturated and saturated lipids present in coffee, corn and barley oils that do not change during roasting. Absorption bands at 2250–1850 cm^−1^ were observed in all matrices but they were significantly more intense on roasted corn and barley and probably contributed to the discrimination between pure coffee and cereal samples. These bands were correlated to the presence of phenolic compounds (ferulic and coumaric acids and their derivatives) bound to non-degraded starch in the cereals. In the spectra of coffee, corn and spent ground coffee, a band at 1745 cm^−1^ was endorsed to the carbonyl stretching vibration of triglycerides. This band was weaker in spectra from roasted coffee husks and barley, which instead exhibited a band at 1715 cm^−1^, assignable to free fatty acids that were originated by the possible degradation of their lipid contents. Bands at 1700–1660 cm^−1^ could be assigned to caffeine, as they are more intense in coffee and coffee husks spectra than in spectra of samples that do not contain (barley and corn) or contain low levels of this compound (spent coffee). On the other hand, the presence of absorption bands at 1650–1400 and 1585–1575 cm^−1^ were related to trigonelline. These bands were observed in coffee and coffee husk spectra but not in spent ground coffee nor in barley or corn spectra. Intense absorption bands in the range 950–700 cm^−1^ were correlated to the presence of starch and useful to discriminate corn and barley from those that do not contain starch (pure and spent coffee and coffee husks). Authors suggested that these wavelength differences could be due to the different types of polysaccharides existent in coffee (arabinogalactans, galactomannans, and cellulose) and its adulterants (starch) [16,22,59]. PCA analysis of the results allowed the definition of pure and adulterated cluster samples, despite some overlapping between roasted corn and barley. The clusters were strongly associated with caffeine, lipids and starch contents present in the different samples. LDA classification models presented recognition and prediction abilities of 100% [15]. PLS regression was also developed aiming at quantifying the adulteration level. The proposed model provided reliable predictions of adulterations at levels as low as 1% *w*/*w* with determination coefficients of 0.99 and 0.98 for the calibration and validation sets, respectively [56]. In later studies, the same research group obtained similar results when assessed the performance of ATR for the same purpose, with the advantage that sample preparation was not necessary [54]. The two acquisition modes were compared using hierarchical models with two levels of PLS discriminant analysis [55]. The first level discriminated between unadulterated and adulterated coffee samples and the second level was able to identify the presence of each adulterant. In addition, data fusion approach was applied in order to combine the data obtain from DRIFT and ATR. With this methodology, the percentage of misclassified samples in the second level models went as low as 0%, and it was possible to identify adulterated coffee samples and to discriminate these adulterants in complex mixtures [55].

### 4.2. Dairy Products

Several researchers have recommended FTIR spectroscopy for applications in dairy products and the AOAC international (Association of Official Analytical Chemists) has approved in 2005, a FTIR method for determination of fat, protein, lactose, and total solid contents in some dairy products [60]. In the last decade, applications of infrared spectroscopy in cheese authentication have diversified and increased. Application of MIR-FT spectroscopy to the analysis of dairy products is presented in Table 2. Some recent applications include determination of geographic origin of cheeses [61], monitoring changes during ripening [62], shelf life and storage conditions, among others [63,64]. Leite et al. [65] explored the potential application of FTIR-ATR coupled with multivariate approaches in order to characterize and detect adulterations in butter cheeses with soybean oil. Butter cheese is a semi-soft cheese, very popular in Germany and Austria for its creamy texture and its butter like flavor. Its production consists of coagulating whole or skimmed milk, followed by desorption of the curd obtained by acidification, washing the dough with water and/or milk, salting, melting the dough with butter oil and finally molding the cheese. Milk is the only source of the fat content in butter cheese and the addition of different fats is considered fraud. In this study, all the butter cheese samples, prepared in duplicate, had the same fat content addition but different percentages of adulterations (ranging from 0 to 100% of substitution) and were performed by replacing the butter oil by soybean oil. The sample with 0% of fat addition was composed only of cheese mass. The authors concluded that a clear differentiation in the cheese spectrum can be observed, especially in the wavelength range of 3600 to 3050 cm^−1^ and 1000 to 400 cm^−1^ when compared to the fat-free sample with the samples to which butter oil and soybean oil were added. Moreover, it was noted a band at 3007 cm^−1^ characteristic of unsaturated fatty acids, indicating the presence of soybean oil in the samples. This band shifted to 3009 cm^−1^ when 20% to 100% of adulteration was present. Another indication of fraud could be the lack of the water band at approximately 3265 cm^−1^ in adulterated cheeses. Additionally, the authors showed that PCA revealed the samples confirming the similarities found in the spectral analysis. For the percentage of soybean oil present in the adulterated samples of butter cheese, PLS regression resulted in good predictions regarding the substitution levels (squared coefficient values (*R*^2^) of 0.997 and 0.998 for calibration and validation, respectively), with low residual errors.

Alkhalf and Mirghani, reported a study to confirm that MIR spectroscopy allied with multivariate analysis has great potential for detection of formaldehyde in cheese [66]. Although human exposure to formaldehyde occurs most commonly by the respiratory and dermal routes, it may also occur by ingestion. Most of the time, formaldehyde in food is a completely natural occurrence. The European Food Safety Authority noted that natural levels of formaldehyde in food widely vary, depending on the type of product. The US Environmental Protection Agency has established a maximum daily dose reference (RfD) of 0.2 mg kg^−1^ body weight per day for formaldehyde. At exposures increasingly greater than the RfD, the potential for adverse health effects increases (including headaches, nausea, drowsiness and allergic skin reactions, among others). In this study, two sets, each of twenty-one samples were prepared using the same type of soft white cheese and formaldehyde was spiked in the range from 0 to 100 mg/100 g in freshly prepared cheese [66]. The spectral response to changes in formaldehyde content was investigated by examination of the correlation and variance spectra. The spectral region used for correlation and cross-validation was defined and included the spectral intervals from 1650–800 cm^−1^. R^2^ value of correlation was 0.986 with average standard error of calibration of 2.24 mg/100 g. The R^2^ of validation found was 0.9662, the standard errors of prediction and standard deviation of the differences for repeatability and accuracy were 4.07 mg/100 g and 4.61, respectively. The authors concluded that the use of MIR spectroscopy data with PLS regression proved to be a highly accurate approach for the determination of minor components such as formaldehyde in cheese samples. Studies of milk and milk products by FTIR-ATR spectroscopic method have been made by many researchers. Another example of research in dairy products by FTIR-ATR spectroscopic method is the work conducted by Sara et al. [61]. The aim of this study was to combine MIR spectroscopy data with chemometric analysis to confirm cheese quality and products according to their manufacturing process. The study utilized ten samples of traditional cheese-making mozzarella located in a Protected Designation of Origin region and ten samples of industrial cheese-making mozzarella in non-Protected Designation of Origin in Italy. Several spectral regions were used, namely, the intervals 2900–2827 cm^−1^ and 1782 to 1705 cm^−1^ (corresponding to fat content in cheese), 1701–1507 cm^−1^ (protein content) and 1200 to 967 cm^−1^ (corresponding to carbohydrates). The traditional buffalo cheese samples showed greater intensity in the spectral bands, which reflected variation in its physicochemical composition, in relation to mozzarella cheeses obtained by industrial processes. In order to discriminate between the two types of buffalo mozzarella, a logistic regression was exploited. The chosen category was the traditional Protected Designation of Origin type of mozzarella, whereas the explanatory variables were absorbance and wavelength. The results suggested the use of MIR spectroscopy with chemometric analysis allowed to discriminate between the two types of mozzarella.

After olive oil, milk is the second food item that is at risk of adulteration [67,68,69]. Adulteration of milk can have economic purposes as in the case of the addition of vegetable proteins, milk of different species, addition of whey and water, not representing any serious risk to health. However, other adulterants are harmful because they cause serious adverse health effects such as the addition of urea, formaldehyde, detergents, ammonium sulfate, boric acid, benzoic acid, salicylic acid, hydrogen peroxide, sugars and melamine [68,70]. A recent study [71] suggested the use of FTIR associated with chemometrics to quantify the cheese whey added in milk, which can be used as a marker of adulteration. Although the use of cheese whey in some products is allowed, particularly in Brazil, it is often used as adulterant for pasteurized, Ultra High-Temperature Pasteurized and dry milk. The detection of this fraud is done through the quantification of caseinomacropeptide, which is a constituent of cheese whey. In this research, three milk types were used, skimmed, semi-skimmed and raw milk. For adulteration of milk samples (*n* = 18), standard caseinomacropeptide was added in 25 mL of milk and subsequently diluted to a final concentration. The infrared spectrum of the adulterated milk samples was performed in the range of 4000–800 cm^−1^. For exploratory analysis of the pre-processed infrared spectra, Principal Component Analysis and Hierarchical Component Analysis were applied before the quantification step. For the multivariate regression the concentrations determined by LC-MS/MS were used as reference values. Algorithms of Partial Least Square Regression, Partial Square Regression and Regression by Minimum Square Support Vector Machine were used and with good regression parameters, which led the authors to conclude that the developed methodology was adequate to detect and quantify the adulteration by adding caseinomacropeptide.

The addition of melamine (2,4,6-triamino-1,3,5-triazine) to milk (liquid or powder) is an adulteration made in order to increase the apparent protein content, causing false readings in the nitrogen determination methods since melamine has a nitrogen mass identical to that of proteins. Melamine is not a natural product and is not approved for direct addition to foods. When melamine is in combination with cyanuric acid, another triazine, it can result in the formation of insoluble melamine cyanurate crystals in the kidneys, causing renal failure [72]. However, melamine is used in the manufacture of certain materials that come into contact with food and therefore the World Health Organization in 2008 considered that the tolerable daily intake is 0.2 mg kg^−1^ of body weight [72]. MIR spectroscopy combined with chemometrics was proposed by García-Miguel et al. [73] for fast determination of melamine and cyanuric acid in infant formula powders (baby milk). In this research, 40 samples of infant formula powder with melamine or cyanuric acid in concentrations ranging from 0.5 µg/L to 20 µg/L were prepared. Five samples were used for the validation model. The spectral area studied was 3600–2800 cm^−1^ and 1750–650 cm^−1^ because this range had the highest association between the spectral data and the sample concentrations. In order to correlate the IR signal with the levels of melamine or cyanuric acid in the infant formula samples Partial Least Squares (PLS1, PLS2) and Principal Component Regression were used. The detection limit (>0.5 µg/L) was below the maximum residue levels established by the Food and Drug Administration (1 mg/kg) [74]. To distinguish between adulterated formulas and non-adulterated samples the Soft Independent Modeling of Class Analogy model was applied with optimum discrimination and good interclass distances between samples. The authors concluded that this model can identify and classify the samples unadulterated and adulterated with melamine and cyanuric acid with a 99% confidence limit.

In a similar approach, Jawaid et al. [75] assessed FTIR-ATR for the determination of different ratios of melamine at various concentrations in milk samples. Initially the interval (4000–650 cm^−1^) was used to study the spectral characteristics of different ratios of melamine at various concentrations. Then, three different selective regions of the MIR spectrum were used to construct Partial Least-Squares calibration model. These models were established for correlating spectral data to melamine concentration with *R*^2^ > 0.99. The region from 840 to 726 cm^−1^ was selected since offered improved regression result when compared to other regions. The limits of detection and quantification of the method were 2.5 ppm and 15 ppm, respectively, which allowed the authors to state that this method is more sensitive compared to the HPLC method used to determine the melamine content in rice and corn flours. Since the IR spectra showed variations in the positions and shapes of peaks between the control and the adulterated milk, Santos et al. [70] have investigated the feasibility of MIR-microspectroscopy to detect and quantify milk adulteration. Samples of milk were taken from local grocery stores and spiked at different concentrations of whey, hydrogen peroxide, synthetic urine, urea and synthetic milk resulting in 310 adulterated samples. The authors found that the spectral changes of MIR-microspectroscopy were almost imperceptible at the lowest levels of adulteration and, in order to extract relevant information for qualitative and quantitative analyses, spectral mathematical transformations (second derivative) were performed. Pattern recognition analysis by Soft Independent Modeling of Class Analogy models showed tight and well-separated clusters allowing discrimination of control samples from adulterated milk with whey (>7.5 g/L), synthetic milk (>0.1 g/L), synthetic urea/urea (>0.78 g/L) and hydrogen peroxide (>0.019 g/L). PLSR models gave prediction errors of 1.91, 0.25, 0.34, 0.05 and 0.014 g/L for whey, urea, synthetic urine, synthetic milk and hydrogen peroxide, respectively. Another example of milk adulteration is the addition of milk of different species. Recently, a quantification of the adulteration of goat milk by cow milk with MIR and Raman spectroscopy combined with multivariate analysis was described by Yaman [64]. Goat’s milk products are adulterated for economic purposes since goat’s milk has a higher price and higher nutritional quality than cow’s milk. Goat’s milk generally has a more nutritious fatty acid profile than cow’s milk fat. Besides, goat’s milk is characterized by having an easier digestion in relation to cow’s milk because it has a higher percentage of short and medium chain fatty acids (C6–C14) and smaller fat globular sizes. In addition, β-carotene, present only in cow’s milk, is the target component for the differentiation of these two milk species from one another [76]. Forty-two milk samples (with 5% increments from 0 to 100%) were prepared from organic full-fat cow and goat milk. FT-MIR and Raman spectroscopy with chemometric techniques were applied in order to detect the presence and quantity of cow milk in mixtures. To extract information, the spectral bands at around 1373, 1454, and 956 cm^−1^ for IR and 1005, 1154, and 1551 cm^−1^ for Raman spectroscopy, respectively, were evaluated. The Raman and IR spectra were correlated with the percentage of adulteration and β-carotene content to develop quantitative models with partial least squares regression. Using Soft Independent Modeling of Class Analogy, results showed that 20% intervals of the mixture could be differentiated barely from other mixtures by MIR spectroscopy; however, they could not find significant discrimination by Raman spectroscopy. The outcome of this research indicated that this study can be enlarged for the mixtures containing different fat content and raw milk samples. In a similar approach, Souhassou et al. [77] reported a study for the detection of camel milk adulteration with cow milk.

### 4.3. Honey

Honey is defined as “the natural sweet substance produced by Apis *mellifera* bees from the nectar of plants, or from secretions of living parts of plants, or excretions of plant-sucking insects on the living parts of plants, which the bees collect, transform by combining with specific substances of their own, deposit, dehydrate, store and leave in the honeycomb to ripen and mature” by the European Union Council Directive from 20 December 2001 devoted to honey (2002) [80]. Sugars are the main constituents of honey, comprising about 95% of honey dry weight. Main sugars are the monosaccharides hexoses, fructose and glucose, which are products of the hydrolysis of the disaccharide sucrose. Besides, about 25 different oligosaccharides have been detected in the composition of honey [81]. FT-MIR has been utilized as a rapid tool for the determination of both geographical and botanical origins of honey and for the unifloral honeys authentication, in particular when used in combination with multivariate data analysis and chemometrics. Moreover, the FTIR spectroscopy has been used to detect adulteration of honey, since it is a serious problem as it causes change in its nutritional and organoleptic qualities having a significant economic impact. Application of MIR-FT spectroscopy to the analysis of honey is presented in Table 3.

In a recent work, Kasprzyk et al. [80] reported a study for rape (Brassica) honey by chemometrics and spectroscopy. Honey is classified as unifloral rape honey, if rape pollen constituted more than 45% of the total amount of pollen grains of nectariferous plants. In this study, a FTIR-ATR spectrum (4000–400 cm^−1^) of each honey sample and rape pollen collected from flowers (reference) was acquired. To analyze the results, these authors developed a large array of chemometric procedures including Detrended Corresponce Analysis, Multidimensional Scaling, Hierarchical Clustering Analysis, and Mahalanobis distance. Discriminate analysis was used to identify rape-honey in a taxonomical discriminant analysis. In addition, the method Classification Tree Analysis, was used in order to allow a clear identification of the spectra characteristics, permitting the authentication rape honey. The authors concluded that these methods may be potentially used for identification of other unifloral honeys [80].

In order to rapidly and accurately differentiate the authenticity and classification of honey, Sahlan et al. [82] developed a method based on ATR-FTIR spectroscopy and chemometric discriminant analysis. For this study, they used a total of 85 samples. Honey samples were grouped in fake honey (adulterated) and real honey. The fake honey samples (*n* = 27) were made by mixing real honey with water, sucrose, and NaHCO_3_. The samples of real honey (*n* = 58), were produced by sting bee *Apis* spp. and by stingless bee *Tetragonula* spp. The content of honey between them has chemical differences, stingless honey has an exceptionally higher content of flavonoids and polyphenols compared to honey produced by *Apis* spp. The potential health benefits of stingless bee honey (SBH) are only recently gaining a lot of attention [83]. Besides, the cost of honey derived from stingless bees is much higher than that of honey derived from other bee species. The wavelengths that best differentiated between real honey and adulterated honey correspond to four regions: 1600–1700 cm^−1^; 1175–1540 cm^−1^; 940–1175 cm^−1^; and 700–940 cm^−1^. For classification of the honey samples, the authors reported significant spectral differences between honey produced by *Apis* spp. and honey produced by stingless bee *Tetragonula* spp. The wavelength range that can best differentiate between them was 1600–1700 cm^−1^. The bands in this region were due to stretching band of carbonyl groups C=O and C=C related to phenolic molecules.

To analyze and authenticate honey samples, important to protect industry and consumers from adulterated honey, Rios-Corripio et al. [84] described the use of ATR-FTIR spectroscopy and multivariate methods to study honey intentionally adulterated with three types of standard sugars (glucose, fructose and sucrose) and also with three types of cheap syrups (corn syrup, inverted sugar syrup, cane sugar syrup). The determination of the type of adulterant was realized by using the Principal Component Analysis method on pure and adulterated (0–100%) honey samples. The calibration and validation models were developed to predict quantitatively the adulteration on the basis of the spectral information mainly in the regions 650–1980 cm^−1^ and 2400–3700 cm^−1^. Partial Least Squares regression was used and showed good predictive capacity of the model employed in this study. Another work [85], involving honey from stingless bees (*Heterotrigona itama*), described the use of FTIR-ATR spectroscopy with chemometric analysis for discrimination of pure and adulterated *H. itama* honey. Standard sugars (fructose, glucose and sucrose) and commercial sugars (corn syrup and cane sugar) were used as adulterants. In this study, after classifying the pure and adulterated samples using PCA and SIMCA method, concentrations of adulterants were predicted using PLSR analysis based on FTIR-ATR spectral data. The authors demonstrated that FT-IR spectroscopy is a method highly reliable for the rapid identification and precise quantification of adulterants in *H. itama* honey [85].

### 4.4. Olive Oil

Olive oil is obtained from the fruits of *Olea europeae* tree and is an extremely important economical product in Mediterranean countries [89]. On average, 3 million tonnes of olive oil are produced around the world every year. European Union is the first producer, consumer and exporter, accounting for almost 2200 million tonnes of world production. The main European Union member states include Spain (63% of production), Italy (17%), Greece (14%) and Portugal (5%), but also France, Slovenia, Croatia, Cyprus and Malta [90,91]. Outside European Union, Morocco, Turkey and Tunisia are also important world production countries with an estimated production of 200, 183 and 120 tonnes for 2018/2019 [92]. European Union has also a leading role on the international market, both as an importer and an exporter of olive oil. The large majority of exports are intra-European Union or to third-countries such as United States, Canada, Brazil, Australia and Japan [91].

There are several categories of olive oils grouped according to its physicochemical and organoleptic quality parameters, as defined by European legislation [93]. Virgin olive oils are exclusively extracted by mechanical or physical methods such as pressure or centrifugation, which do not lead to alterations in the oil, and are subdivided in extra-virgin olive oil, virgin olive oil, ordinary virgin olive oil and lampante olive oil. Extra-virgin olive oil is the highest quality and expensive category, and from the organoleptic point of view, it is a fruity oil with no defects. It differs from virgin olive oil in the acidity level (expressed as oleic acid) that cannot be higher than 0.8% for extra-virgin olive oil, 2.0% for virgin olive oil and 3.3 for ordinary virgin olive oil. Lampante olive oil has lower quality, acidity level higher than 3.3% and it is not fitted for consumption, being used for refining or for technical purposes. Other categories of olive oils include refined olive oil that is obtained from a defected virgin olive oil through refining methods, which do not lead to alterations in the glyceridic structure, blends of refined olive oil and virgin or extra-virgin olive oil and olive pomace oil. Olive pomace is the solid residue left over from the production of virgin olive oil. As it still contains a considerable proportion of oil (5–10%) it can be treated with solvents and further refined [94,95].

Olive oil, particularly extra-virgin olive oil, is the primary source of added fat of the Mediterranean Diet, which takes part since 2013 of the representative list of the UNESCO Intangible Cultural Heritage of Humanity [96]. Apart from the consumption of extra-virgin olive oil, the Mediterranean Diet is also characterized by the high consumption of fruits, vegetables, whole cereals and nuts, medium-high intake of fish, moderate consumption of dairy produces (mainly yogurt and cheese), low intake of meat and saturated fat, and a moderate intake of wine during meals. Many studies recognized the linkage between this diet and the low incidence of cardiovascular diseases in Southern Europe in comparison with Northern Europe and the United States, and the prevalence of chronic diseases such as metabolic syndrome, cancer or neurodegenerative conditions [97,98,99]. The positive effects of Mediterranean Diet on the risk of chronic diseases is considered by several authors as an example of food synergy [99,100]. Nevertheless, several studies also emphasize the benefits of the higher consumption of extra-virgin olive oil [101,102,103,104]. Its high nutritional value are endorsed to its unique fatty acid profile and to the presence of other minor components that are responsible not only to the very distinctive flavor but also have an important role on the stability of the oil and antioxidant capacity. Olive oil is mainly composed by triacylglycerols (98–99%), in which prevail monounsaturated fatty acids such as oleic acid (70–80%), and a very low quantity of polyunsaturated fatty acids. It also contains a variable proportion of free fatty acids, phytosterols, diterpenes and triterpene alcohols, squalene and several antioxidant compounds such as α- and γ-tocopherols, tocotrienols, β-carotene, phenolic compounds, such as, oleuropein, hydroxytyrosol, tyrosol, oleocanthal, phenolic acids and flavonoids, as well as chlorophyls and volatile and aroma compounds [89,105]. Olive oil composition differs among the samples and depends on several factors including the geographic location of production, edaphoclimatic conditions, olive cultivar and degree of fruit ripening [89]. The triacylglycerol composition of olive oil is very similar to that of hazelnut but differs significantly from those of maize, cottonseed, sunflower, soybean and rapeseed oils [89].

In the last decades, there have been an increasing interest and demand for high-quality olive oils, as well as their labeled categories such as Protected Designation of Origin, Protected Geographical Indication, and organic or mono-varietal olive oils [106,107]. Consequently, their marketed price is often very high and these products are susceptible to different kinds of adulteration and mislabeling practices. Quality, purity and composition characteristics of olive oils are highly regulated by several international organizations, such as International Olive Council [92] and European Commission [93], among others. These organizations define the classification criteria, physicochemical and sensorial parameters for quality and purity criteria and their corresponding threshold values, as well as the description of official analytical methods for assessing those parameters [96,97,108,109]. Table 4 summarizes the physicochemical quality and purity parameters, the threshold value for extra-virgin olive oil, the objective of their assessment and corresponding analytical methods. As can be observed, most of the analytical methods are based on the specific determination of a marker compound or family of compounds, and involve laborious and complex sample preparation procedures, followed by chromatography analysis. Therefore, they have several drawbacks, being time-consuming, requiring large volumes of organic solvents that generate toxic wastes and need a strictly reproduction of the standardized procedure in order to achieve the necessary accuracy levels. Moreover, they are not effective in identifying the type of adulterant or low adulteration levels [106,107]. To overcome these limitations it will be essential to develop simpler, robust, sensitive, rapid, and environmentally friendly methodologies, which should be easily adapted to routine analysis in order to evaluate quality, authenticity and possible adulteration of olive oils [106,107,110]. In this way, several analytical approaches have been proposed, including improvement of sample preparation techniques (using for example microwave assisted derivatization reactions and solid phase extractions), chromatographic techniques coupled to mass spectrometry, and spectroscopic techniques (UV-Vis, IR, Raman, fluorescence and NMR) [106,107,110].

MIR spectroscopy coupled to chemometric approaches have been an effective strategy to identify and quantified adulteration of extra-virgin olive oil with refined oils and several types of vegetable and nuts edible oils. Specific location and intensity of IR absorption bands depend on triacylglycerol fatty acid composition, unsaturation degree and corresponding proportion of fatty acids, which differ according to the nature of vegetable oils. These spectral features occur at specific regions, mainly around 3000 cm^−1^, 1740 cm^−1^ and the fingerprint region at 1500–600 cm^−1^ (Table 5). In particular, the fingerprint region is very important since it is highly representative of molecular composition, being used for identification purposes [108,111].

Several studies (Table 6) have been reported using MIR spectroscopy combined with different multivariate analysis models to assess the adulteration of extra-virgin olive oil (EVOO) with various vegetable oils including grape seed, soybean and walnut oils [109,112,113], pumpkin seed oil [114], canola oil [112,115], corn and sunflower oils [116,117], rice bran oil [118], sesame oil [119], peanut and rapeseed oils [120,121] and hazelnut oil [122]. Although all IR spectra of EVOO and each vegetable oil looked very similar at first sight, some important differences regarding the exact location and intensity of the main absorption bands were noteworthy. Therefore, it was crucial to select and optimize the wavenumber regions that yield the smallest misclassification of EVOO and corresponding mixtures of foreign oils. The wavenumbers selection depends on the oil used to produce the EVOO adulteration due to the changes in fatty acid composition and content. For example, for pumpkin seed adulteration studies the selected frequency ranges were 3020–2995 cm^−1^ and 1070–900 cm^−1^ [114], whereas for sesame oil adulteration studies the selected frequency ranges were 3050–2927 cm^−1^, 1517–1222 cm^−1^ and 1207–1018 cm^−1^ [119]. In some of these studies, classification of EVOO and adulterated mixtures was performed using Discriminant Analysis, resulting in 100% accurate classification of pure and adulterated EVOOs. Moreover, for quantification purposes the adulterated samples were divided into calibration, prediction and validated sets, and the performance of PLS and PCR regression algorithms were compared. PLS showed a better performance in all studies, exhibiting higher coefficient of determination and lower root mean square error of calibration and validation.

Didham et al. [123] compared the ability of UV-VIS and ATR-MIR spectroscopy associated to PCA and PLS-DA models to detect and quantify artificial adulteration of EVOO with canola and sunflower oils. Two sets of mixtures were prepared adding the vegetable oils to EVOO (10% to 50% and 0.2% to 1%). Both spectroscopic methods were able to detect levels of adulteration higher than 10%, but fail to detect low levels of adulteration. Uncu and Ozen [124] compared the performance of three spectroscopic techniques (fluorescence, FTIR, and UV-visible) in the detection and quantification of adulteration of fresh olive oils with olive oils from the previous harvest year [124]. Spectra of fresh and adulterated samples were visually compared for the three studied techniques revealing evident differences between UV-vis and fluorescence spectroscopy. On the other hand, no significant differences were observed in the FTIR spectra. Orthogonal Partial Least Square-Discriminant Analysis (oPLS-DA) and Partial Least Squares (PLS) regression techniques were used to distinguish adulterated from non-adulterated oils. oPLS-DA classification models were created using the individual and combined data from the three techniques. All models were able to successfully discriminate fresh and old olive oils and their mixtures with over 90% correction. Moreover, fluorescence and the combination of FT-IR and UV-Vis provided better results in the quantification of adulteration than the two other individual spectroscopic techniques and were also successfully used to predict adulteration levels with high coefficient of determinations for both calibration (0.94 and 0.98) and prediction (0.91 and 0.97) and low error values for calibration (4.22% and 2.68%), and prediction (5.20% and 2.82%) [124].

Due the high complexity of data obtained in food analyses, there has been an increasing request for more accurate pattern classification algorithms. New multivariate classification models have been investigated, including Back Propagation-Artificial Neural Network, Least Square-Support Vector Machine and Continuous Locality Preserving Projections technique coupled with k-Nearest Neighbors on the adulteration of extra-virgin olive oil with peanut and rapeseed oils [120] and hazelnut oil [122].

In a recent study, Xu et al. used FTIR spectroscopy associated with three multivariate calibration algorithms, namely, Linear Discriminant Analysis, Back Propagation-Artificial Neural Network and Least Square-Support Vector Machine to verify the adulteration of EVOO with peanut and rapeseed oils [120]. Remarkable differences were observed among the performance of the three models. Least Square-Support Vector Machine achieved a higher discrimination rate of 92.5% when compared with Linear Discriminant Analysis (85%) and Back Propagation-Artificial Neural Network (82.5%) classification algorithms. The authors concluded that FTIR spectroscopy could provide a rapid detection of adulterated EVOO when combine with a properly selected and accurate multivariate calibration model [120].

FT-MIR has also been broadly used to evaluate the quality and authenticity of olive oils [125,126], as well as to determine its geographic and varietal origin [127,128,129,130]. In this context, several chemometric models have been developed taking in account the fingerprint of different chemical compounds such as triglycerides, fatty acids or steroids [106]. Relevant examples are summarized in Table 6.

In a recent study, Üçüncüoǧlu and Küçük aimed at classifying fresh Turkish virgin olive oils based on cultivar and geographic origin as well as detecting possible chemical differences on lipid structure at the end of twelve month storage, using FTIR coupled with Principal Component Analysis [125]. The method successfully defined the different varieties of olive oil. Furthermore, a fingerprint of the slow-paced oxidation process could also be deduced from the bands at 3008, 2924, 1745 cm^−1^, corresponding to the symmetric and asymmetric stretching vibration of aldehydes, ketones, alcohols and hydroperoxides, which were found to be more intense at the end of the storage time [125]. A research comparing the performance of NIR, MIR spectroscopy and electronic nose (e-nose) on discrimination of different varieties of Turkish olive oils was reported by Jolayemi et al. [127]. Principal Component Analysis and orthogonal partial least square-discriminant analysis models were applied. Correct classification of samples were obtained for NIR and combined MIR and NIR techniques (higher than 90%), in comparison with e-nose technique (82%) that is suggested to be used as a complementary method to human sensory analysis [127]. A total of fifty-five EVOOs from seven Tunisian cultivars were successfully discriminated using MIR and linear discriminant analysis [128]. Using 20 spectral regions, mainly corresponding to vibrations associated with C–H, C–O, C=C (aromatic), and =C–H groups all the samples were correctly classified. Moreover, multiple linear regression was used to detect mixtures of EVOOs from different cultivars with an average validation error below 6% [128].

### 4.5. Wine

Wine is a traditional alcoholic beverage and due to its high economic importance, it is one of the most common beverages subject to fraud and mislabeling. The wine manipulations may be due to changes in intrinsic properties (for example, dilution of wines with water, addition of alcohol, coloring or flavoring substances) or changes in extrinsic properties (for example, fraudulent misrepresentation of the geographical origin) [134]. Wine is mainly composed of water and ethanol (95%). Other components such as glycerol, organic acids, carbohydrates, minerals, volatile compounds, and phenolic compounds, among others, represent <5% [135,136]. The majority of the phenolic constituents found in wine are grape-derived and in red wine, tannins and anthocyanins are the most important phenolic classes. Tannins are responsible for the stabilization of the color and the sensory characteristics of the wines due to their astringent and bitter properties [137]. In white wine, the most important phenolic compounds are the hydroxycinnamic acids and to a lesser extent, the flavan-3-ol monomers [137]. The methods based on the FTIR-ATR technique have a huge application in the wine industry because they have the ability to simultaneously measure several analytes in the same sample at the same time, which makes this technique very attractive for use in both industry and research. The potential of MIR- ATR for the rapid analysis of multiple wine components has been extensively reported. Relevant examples are summarized in Table 7. Different applications of this spectroscopic technique to wine have been published, such as the measurement of phenolic compounds as well the total antioxidant activity of wines [138,139,140], or as a tool for the control of wine maturation time [141,142], the monitoring of sugars, alcohol, and organic acids [138], among others. Furthermore, over the last decades, many studies have shown that MIR spectroscopy combined with multivariate data analysis is a valuable tool both in assessing wine quality and its geographical origin as well its authentication and fraud. In a recent work, Geană et al. [143] executed a feasibility study on the use of MIR-ATR and UV-Vis spectra combined with multivariate statistical tools to classify samples of authentic red wines (*n* = 39), made from different red grape varieties and produced in a single area, Dobrogea region. These wines from Romania were from different years of harvest (from 2009 to 2017). The spectra of all wines showed similar peaks and only minor differences can be observed in specific areas of the spectra. In this work, the authors focused on the 1600–900 cm^−1^ spectral region, and consequently it was selected for statistical analysis since absorptions in this region are due mainly to the stretching and bending vibrations associated with phenolic compounds. In order to classify the wine samples according to the variety and year of harvest, several multivariate analysis tools were used, such as Principal Component Analysis, Partial Least Squares Discriminant Analysis and Linear Discriminant analysis. In this study, the spectral data were directly statistically processed, without any prior pre-treatment. The authors concluded that UV-Vis spectroscopy is more appropriate for varietal discrimination of red wines, while MIR spectroscopy was more efficient for the prediction of wine vintage year since a clear discrimination of aged wines (over six years) was observed. Using a similar approach, Banc et al. [139], assessed the potential of FTIR-ATR to characterize 15 different Romanian wines (white, rosé and red wines), obtained from different authentic, origin-denominated cultivars, and produced in the vintage years 2008–2012. In this study, MIR absorption spectra of each wine were splitted in four regions corresponding to the most important components, phenolics (600–940 cm^−1^), carbohydrates (970–1100 cm^−1^), amino acids and organic acids (1600–1700 cm^−1^). In the last region (2800–3000 cm^−1^), all wines absorbed at the same wave numbers, 2887 and 2931 cm^−1^. Principal Component Analysis and Hierarchical Cluster Analysis was performed in order to identify the specific discrimination factors useful to authenticate the biological (cultivar) and regional origin, as well their sweetness index. The authors showed that the combination of MIR-ATR and chemometric techniques presented in this study allowed almost complete discrimination between samples and might be used as a technique for the discrimination between different red, rosé and white wine varieties.

Recently, MIR was successfully applied for discriminating among different Italian monovarietal red wines (*n* = 110), vintage 2016, based on the relationship between grape variety (*n* = 11) and wine composition, in particular phenolic compounds [140]. The authors selected multivariate tools, Principal Component Analysis, Linear Discriminant Analysis, Soft Intelligent Modelling of Class Analogy, and Support Vector Machine, in order to classify the red wines according to their grape variety. The best result was obtained with the Support Vector Machine model, which achieved an overall correct classification for up to 72.2% of the training set, and 44.4% for the validation set of wines, respectively. Ioannou-Papayianni et al. [144] reported a work using MIR and chemometrics, in order to study the authenticity and uniqueness of the Cypriot traditional wine, “Commandaria” wine that is produced from sun-dried grapes. Different sweet wines were analyzed (*n* = 65) to compare the infrared spectra of 31 Cypriot samples “Commandaria” (19 non-fortified, 12 fortified, fortified meaning that alcohol of grape origin was added during manufacture) and 7 commercial “Commandaria type” (home produced) with that of 27 other sweet wines (3 from Cyprus, 12 from Greece, and 10 from other countries). The spectra were statistically analyzed using multivariate chemometric techniques, involving Principal Component Analysis, Cluster Analysis, Linear and Regularized Discriminant Analysis, and Classification and Regression Trees. The best results were obtained using Principal Component Analysis, and the total correct recognition ability was very high with both Regularized Discriminant Analysis and Classification and Regression models, but the prediction for “Commandaria” was higher using Regularized Discriminant Analysis. The authors concluded that these investigation allowed a nearly correct classification for Commandaria.

Findings reported by Hu et al. [145] showed that MIR and NIR spectroscopy and multivariate chemometric techniques could be used to classify Cabernet Sauvignon wines from different countries. All studied samples (*n* = 540) were from 2010 to 2016 vintages. Cabernet Sauvignon is considered as an ancient and traditional red wine grape variety derived its fame from the south west of France, and in emerging grape growing regions, such as Australia, Chile and China. In this work, the authors observed that through the Tri-step infrared spectroscopy, a spectral technique integrating Fourier transform infrared spectroscopy, second derivative infrared spectroscopy and two-dimensional correlation infrared spectroscopy, it was possible to reveal the main constituents in complicated mixture systems and distinguishing the types and contents of chemical components in highly similar matrices, as the wines investigated here. Combining the spectral characteristics extracted from Tri-step IR analysis and chemometric techniques, authors concluded that Soft Independent Modeling of Class Analogy models correctly classified 97%, 97% and 92% of Australian, Chilean and Chinese Cabernet Sauvignon wines, while the Discriminant Analysis models correctly classified 86%, 85% and 77%, respectively. This work is yet another recent example of the suitability of FTIR and chemometric methods to establish models of geographical origin traceability [145].

## 5. Future Perspectives

This review focused on the recent research on MIR spectroscopy for the analysis of five foodstuffs that are considered more susceptible to fraudulent acts, namely, coffee, dairy products, honey, olive oil and wine. Altogether, the information gathered is very relevant since it embraces the efforts of several academic research groups worldwide, and clearly reveals that the MIR spectroscopy associated with attenuated total reflection acquisition mode and different chemometric tools could be broadly applied to address quality, authenticity and adulteration issues. Nevertheless, the majority of these studies are purely academic. In order to be further applied as a potential analytical tool in official food control procedures, several challenges must be overcome [4,146]. The implementation of validated standard methods are of utmost importance aiming at assuring a high level of reproducibility across different laboratories, equipment and analyses, allowing data to be comparable, which is mandatory in official food control procedures [146]. In this context, validation guidelines are urgently needed to standardize all the steps of method development in MIR spectroscopy. Choosing an adequate source of samples is also essential for method development. The origin of samples should be known, preferably from reputable producers rather than from commercial outlets or markets. Certified reference materials should be used whenever possible [4]. For both method development and validation, it is also imperative to collect a sufficient number of representative samples in order to cover all sample variations and develop a robust model that could be further employed for legal and regulatory purposes. Furthermore, the choice of the most appropriate chemometric methodology is another issue that must be addressed. A plethora of multivariate analyses have been employed for processing the vast collection of spectral data. Some of these software tools are expensive and data analysis require time for the complex statistical treatments and specific abilities to interpret the results.

With the constant change of food industry in the recent decades, the use of new technologies has intensified and plays an increasingly important role in this sector. Big data analytics, IoT (internet of things) and cloud computing, technologies that are all under the umbrella of Industry 4.0 can make a significant difference in the food and beverage sector with regard to compliance and product provenance. Many examples of application of Industry 4.0’s in Food Industry have been reported [147,148,149,150]. As mentioned in this review, food authenticity has recently been a major concern and priority as well as food safety. For example, the IoT technology, a technology-Key Industry 4.0, has proven to be a solution to this concern, since it identifies the product and provide traceability from cultivation to the production chain for food processing [151]. In the future, Industry 4.0 will allow food production systems to produce better food and beverages, make a big leap in terms of productivity improvements, as well as in improving food quality.

## 6. Conclusions

Food adulteration and food authenticity are issues of increasingly high concern to consumers and to all stakeholders involved in the food production and food industry. Nowadays, several physicochemical and instrumental analytical methodologies exist to detect fraud. The vast majority of these methods are based on a target approach focusing on the detection of a specific compound or a family of compounds. These assays are often complex and time-consuming, requiring several steps for sample preparation and highly qualified laboratory technicians, being less suitable for routine or large-scale analyses. Furthermore, as most adulterants are unknown, it is very unlikely that they could be detected using target methodologies [4]. In the last years, with the urgent need to develop new tools, researcher’s attention has been focused on the application of untargeted methodologies to food analyses. Untargeted methods such as spectroscopic (IR, Raman, NMR), hyperspectral imaging and chromatographic techniques (GC-MS and HPLC-MS) provide a molecular fingerprint of the whole food matrix. In particular, MIR spectroscopy has emerged as a potential analytical tool and has been considered an alternative to other more expensive and complex methods, being suitable for implementation in factories during the production process as well as in quality control laboratories. Since it is considered a high-throughput approach, some advantages of this methodology include the speed of analyses, simple or no-sample preparation, fast acquisition of spectra and the possible detection of unexpected adulterants or unexpected deviations to the reference samples [152]. Due to the large and complex spectral data sets provided by MIR spectroscopy, it is mandatory to employ chemometric analyses that allow the extraction of relevant information and the conception of models that could be used to perform exploratory studies and define important features of samples or predict analyses on new samples [27]. In conclusion, we believe that MIR spectroscopy is a rapid and valuable tool that could be useful as a preliminary screening of a commodity, particularly when fast and robust methods are necessary in order to accept or reject a product or a batch before its introduction in a food chain. Nevertheless, further confirmatory analysis may be required using already validated target methods that accomplished the current legislation for official food control.

## Figures and Tables

**Table 1:** Application of FT-MIR spectroscopy to the analysis of coffee.

Product/Reference	Sampling	Wavenumber Range (cm^−1^)	Multivariate Analysis	Aim/Comments
Green coffee beans [46]	ATR	1775–1500 3030–2750	PCA	Assessment of 48 green coffee samples based on their origin (Brazil, Colombia, Ethiopia, Kenya, and Yemen).
Colombian coffee [49]	ATR	4000–650	PCA; PLS-DA	Comparative study of ^1^H-NMR, ATR-MIR, and NIR to discriminate 97 samples of roasted coffee beans from Colombia and other countries.
Green Arabica coffee [50]	Transmission (KBr)	1800–800	SVM	Geographic classification of 20 genotypes
Arabica coffee [47]	Transmission (KBr)	4000–400 1900–800	ANN (RBF)	Geographic and genotype authentication
Roast ground coffee [54]	ATR	4000–700 1735–700	PLS	Simultaneous quantification of four adulterants (coffee husks, spent coffee grounds, barley, and corn)
Roast ground coffee [55]	ATR DRIFT	4000–700	PLS-DA; Data Fusion	Comparing the performance of two acquisition modes (DRIFT and ATR) and employing data fusion (DF) in order to combine both data from DRIFT and ATR.
Roast ground coffee [15,21]	DRIFT	4000–400 3200–700	PCA; LDA	DRIFT as a methodology for simultaneous discrimination between roasted coffee and multiple adulterants was confirmed. LDA classification models presented recognition and prediction abilities of 100%, being able to detect adulteration levels as low as 1 g/100 g.
Roast ground coffee [56]	DRIFT	4000–700	PLS	PLS was employed for quantification of adulterants (pure or mixed) in roasted coffee samples using the DRIFT spectra as chemical descriptors, with adulteration levels ranging from 1% to 66% in mass
Roast ground coffee [57]	ATR	4000–600	PCA; PLS	Identify adulteration in roasted and ground coffee by addition of coffee husks
Roast ground Arabica coffee [58]	ATR	4000–525	PCA; PLS	DSC and FTIR coupled with PCA are able to discriminate adulterated from unadulterated samples of coffee by corn
Arabica and Robusta coffee [59]	ATR	4000–600 1500–2000 3000–2750	PLS	Construction of a PLS calibration model to determine the Robusta content in Arabica coffee blends with 9.2 wt% accuracy
Green Arabica coffee [22]	ATR DRIFT	4000–700	PCA; LDA	Discrimination between defective and non-defective Arabica green coffee beans
Green Arabica coffee [51]	Transmission (KBR) ATR; DRIFT	4000–700	PCA; HCA	Comparison of three IR sampling techniques to discriminate between defective and non-defective green coffee beans
Green Arabica coffee [52]	DRIFT	3100–600	PCA; LDA	Discrimination of defective and non-defective roasted coffee beans
Green Arabica coffee [53]	ATR DRIFT		PCA; Elastic Net algorithm	Comparing the performance of MIR and NIR to discriminate between defective and non-defective roasted coffees
Arabica coffee [38]	ATR	4000–600	PCA, PLS-DA	Prediction of cup quality of coffees subjected to different roasting degrees

ANN: Artificial Neural network; DA: Discriminant Analysis; HCA: Hierarchical Cluster Analysis; LDA: Linear Discriminant Analysis; MLR: Multiple Linear Regression; PCA: Principal Component Analysis; PLS: Partial Least Squares; PLS-DA: Partial Least Squares-Discriminant Analysis; SVM: Support Vector Machine.

**Table 2:** Application of FT-MIR spectroscopy to the analysis of dairy products.

Product/Reference	Sampling	Wavenumber Range (cm^−1^)	Multivariate Analysis	Aim/Comments
Cheddar cheese [60]	ATR	1800–900	PLSR; SIMCA	Characterization of cheese ripening and flavour, and classification of cheese samples based on their age
Butter cheeses [65]	ATR	3600–3050; 1000–400 and 3007	PCA; PLS	Quantification of the adulteration in butter cheeses with soybean oil
Butter cheeses [65]	ATR	3600–2750; 1800–625	PCA; PLSR	Identification and quantification adulterants in butter cheeses
White cheese [66]	ATR	1650–800	PLSR	Determination of minor components (formaldehyde) in cheese samples
Buffalo cheese [61]	ATR	3000–2800; 1700–1500; 1500–900	LR	Authentication of cheese quality and classification of products according to their manufacturing process
Bovine milk [71]	ATR	4000–800	PCA; HCA; PLS; LS-SVM	Quantification of the adulteration in different types of milk by Cheese serum
Infant formula powders [73]	ATR	3600–2800; 1750–650	PLSR; SIMCA	Quantification of melamine and cyanuric acid
Milk (liquid and powder) [75]	ATR	3000–3633; 100–1630; 806	PLSR	Determination of melamine in dairy milk
Milk [70]	ATR	3500–3300; 1640–1500	SIMCA; PLSR	Determination of several potential adulterants
Goat milk [64]	ATR	1373; 1454; 956	SIMCA; PLSR	Detection and quantification of cow milk in goat milk
Goat milk [76]	ATR	3000–950	PLS-DA	Characterization of milk samples according to different goat breeds
Camel milk [77]	ATR	3000–920	PLSR	Adulteration of camel milk by addition of cow milk
Milk [69]	ATR	1630–1680 1510–1570	PCA PLS-DA	Authentication of reconstituted raw milk
Milk [78]	ATR	1800–920	PLSR	Adulteration of raw milk with addition of sucrose
Butter [79]	Transmission; ATR	3910–710	PLS-DA	Adulteration of butter with mutton fat

HCA: Hierarchical Cluster Analysis; LS-SVM: Least Square Support Vector Machine; LDA: Linear Discriminant Analysis; LR: Linear Regression; PCA: Principal Component Analysis; PLS: Partial Least Squares; PLSR: Partial Least Squares Regression; PLS-DA: Partial Least Squares-Discriminant Analysis; SIMCA: Soft Independent Modeling of Class Analogy; SLLE: Supervised Locally Linear Embedding.

**Table 3:** Application of FT-MIR spectroscopy to the analysis of honey.

Products/Reference	Sampling	Wavenumber Range (cm^−1^)	Multivariate Analysis	Aim/Comments
Unifloral honey (Polish rape honeys) [80]	ATR	4000–500	DCA; MDS; MD-DA; CTA; HCA	Authentication of rape (Brassica) honey; potential identification of other unifloral honeys.
Real Honey (samples produced by *Apis* spp. and *Tetragonula* spp.) and fake honey [82]	ATR	Authentication (1600–1700; 1175–1540; 940–1175; 700–940) Classification (1600–1700)	DA	Discrimination between real honey and fake honey and classification between honey from *Apis* spp. and stingless bee *Tetragonula* spp.
Honey samples from Mexico [84]	ATR	850–1200	PCA; PLS	Discrimination of the type of adulterant contained in honey
Honey harvested in Malaysia produced by stingless bees (*Heterotrigona itama*) [85]	ATR	1180–750	PCA; SIMCA; PLSR	Detection and quantification of adulterants in honey from *H. itama*
Honey samples coming from Turkey [86]	ATR	4000–600	GILS; PLS	Determination of honey adulteration in different botanical and geographical origins
Unifloral honey samples [87]	ATR	complete spectral region (4000–600) with emphasis on the fingerprint region from 1800 to 700	PCA, PLSR PLS-DA coupled with ROC analysis	Rapid and reliable determination of nine unifloral honey types
Anatolian honey samples and adulterated honey [88]	ATR	1800–750	PCA and HCA	Discrimination of honey samples from different botanical origins and differentiate inauthentic honey samples from the natural ones

CTA: Classification Tree Analysis; DA: Discriminant Analysis; DCA: Detrendal Correspondence Analysis; GILS: Genetic-Algorithm-based inverse Least Squares; HCA: Hierarchical Cluster Analysis; MD-MA: Mahalanobis Distance- Discriminant Analisys; MDS: Multidimensional Scaling; PCA: Principal Component Analysis; PLS: Partial Least Squares; PLS-DA: Partial Least Squares-Discriminant Analysis; PLSR: Partial Least Squares Regression; ROC: Receiver Operating Characteristic; SIMCA: Soft Independent Modeling of Class Analogy.

**Table 4:** Quality and purity criteria established by International Olive Council [92] and European Commission [93].

		Threshold Value for Extra-Virgin Olive Oil	Analytical Method (Reported Results)	Elucidation of Criteria
Quality Criteria	Free fatty acids	≤0.8	Acid-base titration (% of oleic acid)	Indicative of TAGs enzymatic hydrolysis during manufacturing or storage; Higher values are correlated with worse olive quality or processing of unhealthy olives.
	Peroxide Value	≤20.0	Iodometric titration (meq O_2_/Kg oil)	Indicative of the initial oxidation state of olive oil;
	Ultraviolet absorption	≤2.50 (K232) ≤0.22 (K270) ≤0.01 (ΔK)	UV spectrophotometry at 232 and 270 nm	Indicative of the presence of oxidation products formed during refining process and higher oxidation states.
	Fatty acids alkyl esters	≤35	Isolation by silica-gel column chromatography and subsequent GC-FID analysis (mg/Kg oil)	Indication of health condition of olives and storage conditions before processing. Reported to be a relevant criterion for detecting adulteration with low quality olive oil;
Purity Criteria	Fatty acid composition	Myristic ≤ 0.03 Palmitic 7.50–20.00 Palmitoleic 0.30–3.50 Heptadecanoic ≤ 0.40 Heptadecenoic ≤ 0.60 Stearic 0.50–5.00 Oleic 55.00–83.00 Linoleic 2.50–21.00 Linolenic ≤ 1.00 Arachidic ≤ 0.60 Eicosenoic ≤ 0.50 Behenic ≤ 0.20 Lignoceric ≤ 0.20	GC-FID analysis after a previous methylation reaction (% m/m methyl esters)	Indicative of the presence of foreign oils
	*Trans* fatty acid content	*Trans*-oleic acid ≤ 0.05 *Trans*-linoleic + *trans*-linolenic acids ≤ 0.05	GC-FID analysis after a previous methylation reaction (% m/m *trans*-methyl esters)	Indicative of adulteration with hydrogenated seed oils or low quality olive oil
	ΔAECN42 *	|≤ 0.20|	HPLC-RI and GC-FID	Indicative of adulteration with unsaturated oils
	Sterols	Cholesterol ≤ 0.5 Brassicasterol ≤ 0.1 Campesterol ≤ 4.0 Stigmasterol < campesterol δ-7-stigmastenol ≤ 0.5 Apparent β-sitosterol ≥ 93	Several analytical steps: (i) Saponification of olive oil; (ii) Isolation by TLC; (iii) trimethylsilyl derivatization; (iv) GC-FID (% total sterols)	Indicative of the presence of foreign oils
	Total sterols content	≥ 1000	Same as determination of sterols (mg/Kg)	Indicative of the presence of foreign oils
	Triterpene alcohols	Erythrodiol + uvaol ≤ 4.5	Same as determination of sterols (% total sterols)	Indicative of the presence of pomace oil as well as grape seed oil
	Wax content	≤ 150	Isolation by silica-gel column chromatography and subsequent GC analysis (mg/Kg oil)	Indicative of the presence of pomace oils
	Total aliphatic acids content	Total aliphatic acid content is used in combination with other parameters to distinguish the presence of lampante or pomace oils **	Same as determination of wax content (mg/Kg oil)	Indicative of the presence of lampante and pomace oils
	Stigmastadienes	≤ 0.05	Preparative chromatography followed by GC-FID (mg/Kg oil)	Indicative of the presence of refined oils
	2-glycerylmonopalmitate	If C16:0 ≤ 14.00%; 2P < 0.9% If C16:0 > 14.00%, 2P ≤ 1.0%	Several analytical steps: (i) enzymatic hydrolysis of TAGs; (ii) silica gel chromatography; (iii) trimethylsilyl derivatization; (iv) GC-FID (%)	Indicative of the presence of refined oils

* AECN42: Maximum difference between the actual and theoretical ECN 42 triacylglycerol content (%). ** When the oil has a wax content between 300 mg/kg and 350 mg/kg it is considered a lampante virgin olive oil if the total aliphatic alcohol content is < 350 mg/kg or the erythrodiol + uvaol content is < 3.5%.; When the oil has a wax content between 300 mg/kg and 350 mg/kg it is considered a crude olive pomace oil if the total aliphatic alcohol content is > 350 mg/kg and the erythrodiol + uvaol content is > 3.5% [92].

**Table 5:** Main MIR absorption band assignments for extra-virgin olive oil [108,111].

Wavenumber (cm^−1^)	Functional Group	Type of Vibration
3005	C–H	*cis*=C–H stretching
2955	–CH_3_	Asymmetrical stretching
2924	–CH_2_	Asymmetrical stretching
2855	–CH_2_ and CH_3_	Symmetrical stretching
1746	C=O	C=O stretching (ester groups of TGAs)
1653	C=C	*cis*–CH=CH- stretching
1462–1377	C–H	–CH_3_ and –CH_2_ bending
1162	C–O	C–O stretching (ester groups)
990–960	C–H	*trans*=C–H bending

**Table 6:** Application of FT-MIR spectroscopy to the analysis of olive oils.

Product/Reference	Sampling	Wavenumber Range (cm^−1^)	Multivariate Analysis	Aim/Comments
Australian commercial EVOO [123]	ATR	4000–650	PCA; PLS-DA	Identification and quantification of vegetable oils (canola and sunflower) to olive oil (artificial adulteration)
Olive oils from Turkey (harvested in 2016 and 2015) [124]	ATR	4000–650	PLS-DA; oPLS-DA	Detection of adulteration of fresh olive oils with old olive oils
Brazilian commercial EVOO [112]	ATR	3200–650	PLS	Identification of EVOO adulterated with different vegetable oils (soybean, sunflower, corn, and canola oil) at different levels (1 to 80%, *v*/*v*).
EVOO [120]	ATR	4000–700	LDA; BP-ANN; LS-SVM	Adulteration of EVOO with pure peanut oil and pure rapeseed oil. LS-SVM showed the best performance.
Italian and Greek EVOO [122]	ATR	4000–550	CLPP	Development of a novel continuous statistic model to rapidly detect adulteration of olive oil with hazelnut oil
Commercial EVOO [109,114,115,116,118,119,131]	ATR	3018–3002 and 1200–1000 (grape seed and soybean oils); 3029–2954 and 1125–667 (walnut oil); 3020–2995 and 1070–900 (pumpkin seed oil); 3028–2985 and 1200–987 (canola oil); 3027–3000, 1076–860 and 790–698 (corn oil); 3025–3000 and 1400–985 (sunflower oil); 3050–2927, 1517–1222 and 1207–1018 (sesame oil); 3020–3000 and 1200–900 (rice bran oil)	DA; PLS; PCR;	Adulteration of EVOO with grape seed, soybean, and walnut, pumpkin seed, canola, corn, sunflower, sesame and rice bran oils
Commercial EVOO [121]	ATR	1800–650; 3000–2800	PLS-DA; MC-UVE	Adulteration of olive oil with peanut oil. PLS-DA model using the variables selected by the modified MC-UVE provided 97.6% accuracy, and 100% classification rate
Commercial EVOO [113]	ATR	4000–400	-	Adulteration with soybean oil; Changes in oil in response to high temperatures
EVOO from Italy, Greece, Spain and China [117]	ATR	4000–650	PCA; PLS; SLLE	Adulteration with camellia, soybean sunflower and corn oils (1 to 90%). SLLE gave satisfactory results.
Italian commercial EVOO [132]	ATR	4000–700	PLS	A multi-stage strategy was developed as a powerful tool for monitoring the purity of EVOO and performing qualitative and quantitative determinations of adulterants.
Turkish olive oils (including “Ayvalık” and “Memecik” cultivars) [125]	ATR	4000–400	PCA	Discrimination of VOO by cultivar, geographic origin and storage time
Turkish olive oils (Ayvalik, Memecik, and Erkence cultivars) [127]	ATR	4000–700 (3090–2750 and 1874–700)	PCA oPLS-DA	Comparison of the discriminant abilities of NIR, MIR, and e-nose on authentication of different varieties of Turkish olive oil.
EVOO from seven Tunisian cultivars [128]	ATR	4000–600	LDA MLR	Classification of Tunisian EVOO according to their cultivar
Moroccan virgin olive oils [126]	ATR	4000–600	PLS-DA	Discrimination of four commercial olive oil grades
Italian monovarietal EVOO [133]	ATR Transmission (NIR)	4000–700	PCA LDA, SIMCA	Classification of Italian EVOO according to the cultivar. NIR and MIR techniques were compared giving similar results
Croatian EVOO [129]	ATR	4000–600	PCA HCA	Geographic classification of 48 EVOO
EVOO from Italy (Sabina PDO) and other countries [130]	ATR (MIR) Transmission (NIR)	4000–630	PLS-DA SIMCA	Comparison of NIR and MIR spectroscopy. NIR provided better predictions than MIR

EVOO: Extra-virgin olive oil; BP-ANN: Back Propagation Artificial Neural Network; CLPP: Continuous Locality Preserving Projection; DA: Discriminant Analysis; HCA: Hierarchical Cluster Analysis; kNN: k-Nearest Neighbor; LS-SVM: Least Square Support Vector Machine; LDA: Linear Discriminant Analysis; MC-UVE: Monte Carlo Uninformative Variable Elimination; MLR: Multiple Linear Regression; oPLS: orthogonal Partial Least Squares; PCA: Principal Component Analysis; PLS: Partial Least Squares; PLS-DA: Partial Least Squares-Discriminant Analysis; SIMCA: Soft Independent Modeling of Class Analogy; SLLE: Supervised Locally Linear Embedding.

**Table 7:** Application of FT-MIR spectroscopy to the analysis of wine.

Product/Reference	Sampling	Wavenumber Range (cm^−1^)	Multivariate Analysis	Aims/Comments
Romanian red wines [143]	ATR	1600–900 Coupled with UV-Vis (250–600 nm)	PCA; PLS-DA; LDA	Comparison of UV-vis and FTIR spectroscopy for discrimination and classification of red wines; UV-Vis spectroscopy is more appropriate for varietal discrimination while FT-IR spectroscopy was more appropriate for vintage year prediction
White, rosé and red Romanian wines [139]	ATR	1800–600	PCA; HCA	Method able to discriminate each wine category as a consequence of their biological (cultivar) specificity.
Italian mono varietal red wines (11 grape varieties) [140]	ATR	1500–700	PCA; DA; SVM; SIMCA	Evaluation of quality and authentication of red wines; A peculiar MIR pattern for some Italian grape cultivars was observed. The study of the effect of other variables such as vintage will be done in the future
Sweet wines from Cyprus and other countries [144]	Transmission (KBr); ATR	1900–750	PCA; CA; LDA; CART	Diferentiation of Cypriot traditional sweet wine “Commandaria” from other sweet wines from various countries and of Cypriot provenance.
Cabernet Sauvignon wines from Australia, Chile and China [145]	Transmission (KBr); ATR	1750–1000 Coupled with NIR (4555–4353)	PCA; SIMCA; DA	Authenthication and geographical origin traceability

CART: Classification and Regression Trees; DA: Discriminant Analysis; HCA: Hierarchical Cluster Analysis; LDA: Linear Discriminant Analysis; PCA: Principal Component Analysis; PLS: Partial Least Squares; PLS-DA: Partial Least Squares-Discriminant Analysis; SIMCA: Soft Independent Modeling of Class Analogy; SLLE: Supervised Locally Linear Embedding; SVM: Support Vector Machine.
